# Altered Small-World Efficiency of Brain Functional Networks in Acupuncture at ST36: A Functional MRI Study

**DOI:** 10.1371/journal.pone.0039342

**Published:** 2012-06-22

**Authors:** Bo Liu, Jun Chen, Jinhui Wang, Xian Liu, Xiaohui Duan, Xiaojing Shang, Yu Long, Zhiguang Chen, Xiaofang Li, Yan Huang, Yong He

**Affiliations:** 1 Department of Radiology, Guangdong Provincial Hospital of Traditional Chinese Medicine, Guangdong, China; 2 State Key Laboratory of Cognitive Neuroscience and Learning, Beijing Normal University, Beijing, China; University of Regensburg, Germany

## Abstract

**Background:**

Acupuncture in humans can produce clinical effects via the central nervous system. However, the neural substrates of acupuncture’s effects remain largely unknown.

**Results:**

We utilized functional MRI to investigate the topological efficiency of brain functional networks in eighteen healthy young adults who were scanned before and after acupuncture at the ST36 acupoints (ACUP) and its sham point (SHAM). Whole-brain functional networks were constructed by thresholding temporal correlations matrices of ninety brain regions, followed by a graph theory-based analysis. We showed that brain functional networks exhibited small-world attributes (high local and global efficiency) regardless of the order of acupuncture and stimulus points, a finding compatible with previous studies of brain functional networks. Furthermore, the brain networks had increased local efficiency after ACUP stimulation but there were no significant differences after SHAM, indicating a specificity of acupuncture point in coordinating local information flow over the whole brain. Moreover, significant (*P*<0.05, corrected by false discovery rate approach) effects of only acupuncture point were detected on nodal degree of the left hippocampus (higher nodal degree at ACUP as compared to SHAM). Using an uncorrected *P*<0.05, point-related effects were also observed in the anterior cingulate cortex, frontal and occipital regions while stimulation-related effects in various brain regions of frontal, parietal and occipital cortex regions. In addition, we found that several limbic and subcortical brain regions exhibited point- and stimulation-related alterations in their regional homogeneity (*P*<0.05, uncorrected).

**Conclusions:**

Our results suggest that acupuncture modulates topological organization of whole-brain functional brain networks and the modulation has point specificity. These findings provide new insights into neuronal mechanism of acupuncture from the perspective of functional integration. Further studies would be interesting to apply network analysis approaches to study the effects of acupuncture treatments on brain disorders.

## Introduction

Acupuncture, which utilizes fine needles to pierce through specific anatomical points (called “acupoints”), has been extensively used in traditional Chinese medicine and has emerged as an important modality of complementary and alternative therapy to Western medicine [1], [2]. Many studies have demonstrated that acupuncture plays an important role in relieving pain and anesthetizing patients for surgery [3], [4], [5]. Therefore, it is vital and necessary to explore the underlying biological mechanisms of acupuncture.

Recently, researchers have begun to utilize blood oxygenation level-dependent functional MRI (fMRI), a non-invasive imaging technique mapping brain function, to investigate biological mechanisms underlying the acupuncture therapy. Several fMRI studies have shown that acupuncture stimulation is associated with extensive alterations of brain activity [6], [7], [8], [9], [10], [11]. In particular, research has shown that acupuncture stimulation produces brain activation in several regions of the limbic system, such as the cingulate cortex and insula [6], [7], [8], [9], [10], [11]. However, other fMRI studies showed deactivation in these regions [7], [9], [10], [12], [13], [14], [15], [16]. The results of these studies are inconsistent in regards to the responses of the limbic system to acupuncture stimulation.

Traditional Chinese medicine training on the acupuncture practice supports the concept of acupoint specificity, which typically states that a particular acupuncture point has specific functional effects on the target organ systems. Several previous fMRI studies have indicated that acupuncture at specific acupoints can modulate the brain activity of disease-related neuromatrix [17], [18], [19]. For example, Li et al. [19] showed that acupuncture at traditional “vision-related” acupoints elicited neuronal activity predominantly in the visual cortex. However, other fMRI studies reported that multiple brain regions were activated by such a stimulus [20], [21], [22]. The specificity of acupuncture stimulation remains to be further elucidated.

Despite extensive research on acupuncture-related changes in regional brain activities, very few studies have yet investigated the functional architecture of whole-brain connectivity networks in acupuncture. Currently, there are several different connectivity approaches in studying functional brain networks, such as regional homogeneity (ReHo) [23], seed-based connectivity analysis [24] and independent component analysis (ICA) [25]. Although these connectivity methodologies have been successfully applied to map brain networks from different perspectives and revealed disease-related alterations, they can not capture the topological architecture of brain’s functional connectivity networks (i.e., connectome) (we will return this issue in the discussion). By contrast, graph theoretical approaches allow us to map functional connections among all the brain units simultaneously and to study the underlying topologically organizational principles (e.g., network efficiency and hubs) governing the connectivity networks. Specially, graph theoretical approaches enable us to explore how the entire assemblages of the connectivity networks respond to different external stimulations such as acupuncture. Given that acupuncture is typically thought to modulate and balance the brain activity from global rather than local levels [26], [27], the current study therefore exclusively employed graph theoretical approaches to study how acupuncture affects the topological architecture of whole-brain functional brain networks. Specifically, we focused on small-world organization [28], a consistently observed organizational principle in functional brain networks [29], [30], [31], [32], [33], [34]. The small-wordness is attractive for the characterization of brain function because it not only supports both segregated and integrated information processing but also maximizes the efficiency while minimizing wiring costs [35]. However, no studies reported acupuncture-related changes in small-world properties of whole-brain functional networks.

**Figure 1:**
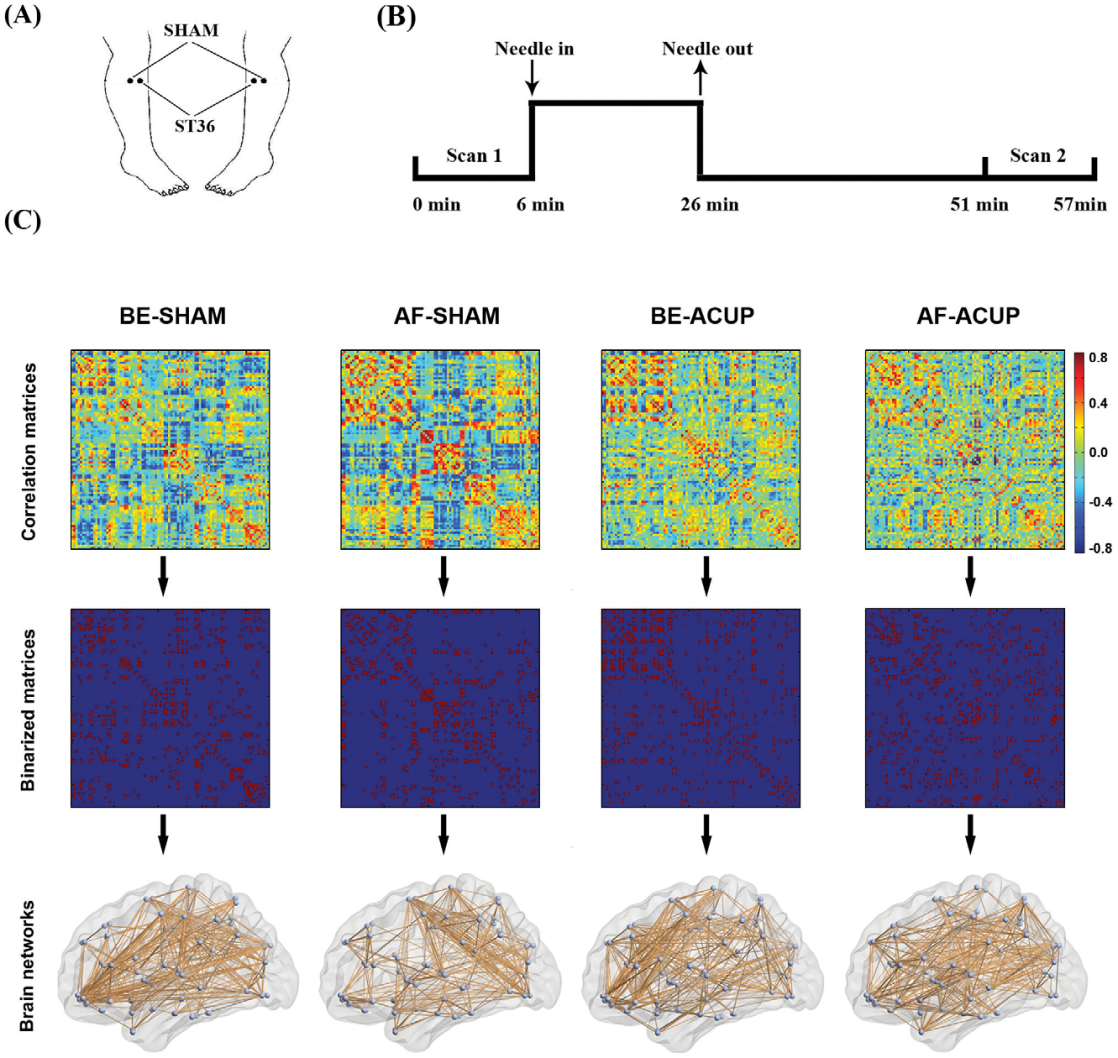
A schematic illustration of the design paradigm and brain network construction. (A), The points of stimulation used in the present study: verum acupuncture at ST36 (ACUP) and sham points (SHAM); (B), Two scans were performed before and after the stimulation at both ACUP and SHAM, which were used to construct brain networks, respectively. (C) Top, under each condition, a correlation matrix was obtained for each subject by calculating inter-regional Pearson correlation coefficient of mean time series among 90 regions; Middle, these correlation matrices were further converted into binary versions (i.e., adjacency matrices) by applying a thresholding procedure such that the elements were set to 1 if their absolute correlation coefficients were larger than a predefined threshold and 0 otherwise; Bottom, the obtained binary matrices could be finally represented as networks or graphs that were composed of brain nodes and edges.

Given that functional connectivity between different brain areas could be modulated by acupuncture [6], [36], we hypothesize that the small-world properties of brain functional networks would be altered after acupuncture. To test this hypothesis, we used fMRI data to construct brain functional networks of ninety brain regions (Table 1) in acupuncture and examined their topological properties such as small-world attributes and hub regions, followed by group comparisons between the acupuncture at acupoints and sham points, and before and after acupuncture. In this study, we selected the acupuncture point Zusanli (ST 36, ACUP) (Fig. 1) because it is the most frequently used acupuncture point in Chinese acupuncture, especially for treating pain, hypertension, gastrointestinal and other physiological dysfunctions [37], [38]. Sham point (SHAM) stimulation was devised with needling at nonmeridian points (2–3cm away from ST36) with the same acupuncture method used in the acupoints.

**Table 1:** Regions of interest (ROIs).

Index	Regions	Abbr.	Index	Regions	Abbr.
1,2	Superior frontal gyrus, dorsolateral	SFGdor	47,48	Middle frontal gyrus, orbital part	ORBmid
3,4	Middle frontal gyrus	MFG	49,50	Inferior frontal gyrus, orbital part	ORBinf
5,6	Inferior frontal gyrus, opercular part	IFGoperc	51,52	Superior frontal gyrus, medial orbital	ORBsupmed
7,8	Inferior frontal gyrus, triangular part	IFGtriang	53,54	Gyrus rectus	REC
9, 10	Rolandic operculum	ROL	55,56	Insula	INS
11,12	Supplementary motor area	SMA	57,58	Anterior cingulate and paracingulate gyri	ACG
13,14	Superior frontal gyrus, medial	SFGmed	59,60	Median cingulate and paracingulate gyri	DCG
15,16	Cuneus	CUN	61,62	Posterior cingulate gyrus	PCG
17,18	Lingual gyrus	LING	63,64	Parahippocampal gyrus	PHG
19,20	Superior occipital gyrus	SOG	65,66	Temporal pole: superior temporal gyrus	TPOsup
21,22	Middle occipital gyrus	MOG	67,68	Temporal pole: middle temporal gyrus	TPOmid
23,24	Inferior occipital gyrus	IOG	69,70	Olfactory cortex	OLF
25,26	Fusiform gyrus	FFG	71,72	Hippocampus	HIP
27,28	Superior parietal gyrus	SPG	73,74	Amygdala	AMYG
29,30	Inferior parietal, but supramarginal and angular gyri	IPL	75,76	Caudate nucleus	CAU
31,32	Supramarginal gyrus	SMG	77,78	Lenticular nucleus, putamen	PUT
33,34	Angular gyrus	ANG	79,80	Lenticular nucleus, pallidum	PAL
35,36	Precuneus	PCUN	81,82	Thalamus	THA
37,38	Paracentral lobule	PCL	83,84	Precental gyrus	PreCG
39,40	Superior temporal gyrus	STG	85,86	Calcarine fissure and surrounding cortex	CAL
41,42	Middle temporal gyrus	MTG	87,88	Postcentral gyrus	PoCG
43,44	Inferior temporal gyrus	ITG	89,90	Heschl gyrus	HES
45,46	Superior frontal gyrus, orbital part	ORBsup			

The regions are listed in terms of a prior template of Anatomical Automatic Labeling atlas (57). Regions in the left and right hemispheres are indexed by odd and even numbers, respectively.

## Results

### Acupuncture Sensation

None of the subjects experienced sharp pain after acupuncture. The prevalence of various acupuncture sensations was expressed as the percentage of individuals in the group that reported the given sensations. The statistical analysis revealed no difference between the ACUP and the SHAM groups in regards to the prevalence of acupuncture sensations (*P*>0.05). There was no significant difference in the pain intensity measured by the VAS between ACUP and SHAM groups (*P*>0.05).

### Global Properties of Functional Brain Networks

#### Small-worldness

**Figure 2:**
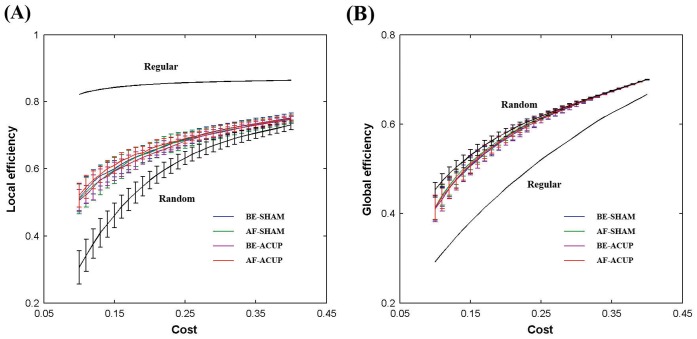
The local and global efficiency of random, regular and actual functional brain networks as a function of cost. The brain networks under each condition showed higher local efficiency than the matched random networks (A) and higher global efficiency than the matched regular networks (B) at the whole cost range between 0.1 and 0.4 used in the present study. Thus, the brain networks under each condition exhibited small-world properties. The brain networks were also found to be economical because both the local and global efficiency were much higher than the required cost.

In the present study, we constructed four brain networks for each participant under each of the four conditions: before stimulation at SHAM (BE-SHAM), after stimulation at SHAM (AF-SHAM), before stimulation at ACUP (BE-ACUP), and after stimulation at ACUP (AF-ACUP). We found that the local efficiency was higher in the regular networks than that in the corresponding random graphs (Fig. 2A), but the global efficiency was higher in the random graphs than that in the corresponding regular networks (Fig. 2B). Furthermore, we found that the efficiency curves of actual brain networks located between the curves of the random and regular graphs in a wide range of cost under each condition, suggesting small-world architectures in the brain functional networks. In addition, all the networks exhibited an economical behavior since both local and global efficiency rose much faster than the required wiring cost (Fig. 2). For example, at approximately 15% wiring cost, the functional brain networks reached local and global efficiency of approximately 50%. These findings were in accordance with previous human brain structural and functional networks studies [39].

#### Point and stimulation effects

**Figure 3:**
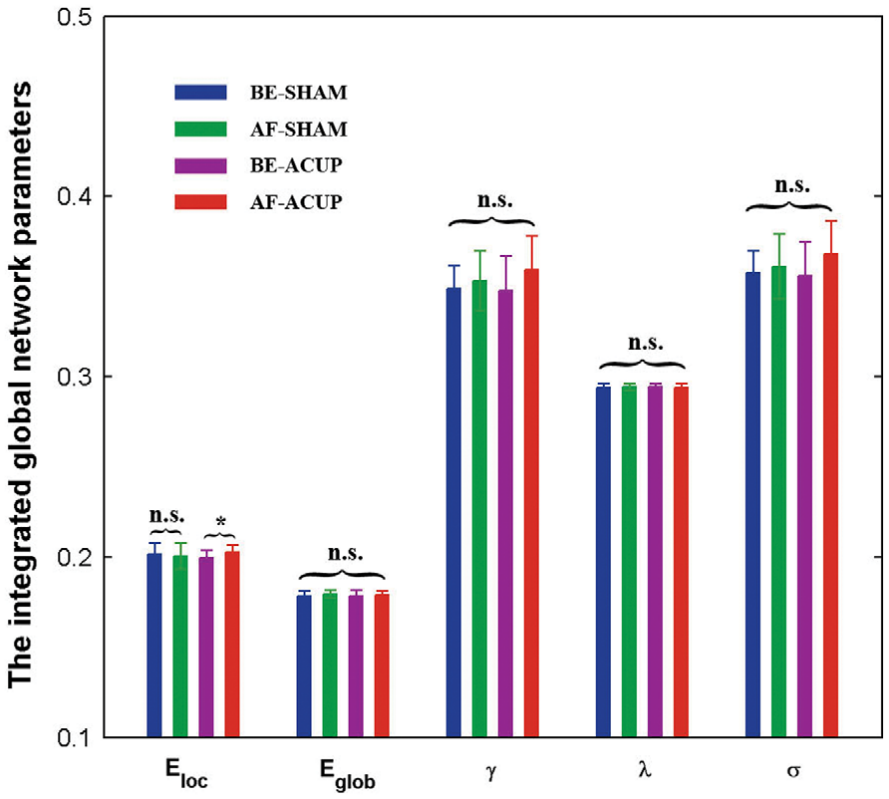
Between-condition differences in the integrated global network parameters. 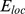
, 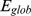, 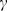, 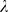 and 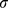 denote the local efficiency, global efficiency, normalized local efficiency, normalized global efficiency, and efficiency-based small-worldness, respectively. Note that the local efficiency (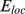) was greater after acupuncturing ST36, but not in the case of SHAM. n.s., non-significant, *, *P*<0.05.

To determine point- and stimulation-related differences, we performed two-way repeated-measures ANOVA using integrated measures (i.e., AUCs). Neither the point effect nor the stimulation effect was significant on any of the five global network parameters (all *P*>0.05). However, we observed a significant point–stimulation interaction on local efficiency (F(1,17)  = 5.66, *P*  = 0.03, Fig. 3). Further paired t-test analysis indicated that this interaction resulted from significantly larger local efficiency (t(17)  = 2.76, *P*  = 0.01) at AF-ACUP when compared to BE-ACUP, but non-significant differences between AF-SHAM and BE-SHAM (t(17) = −0.75, *P*  = 0.46).

### Regional Nodal Degree

#### Network hubs

**Figure 4:**
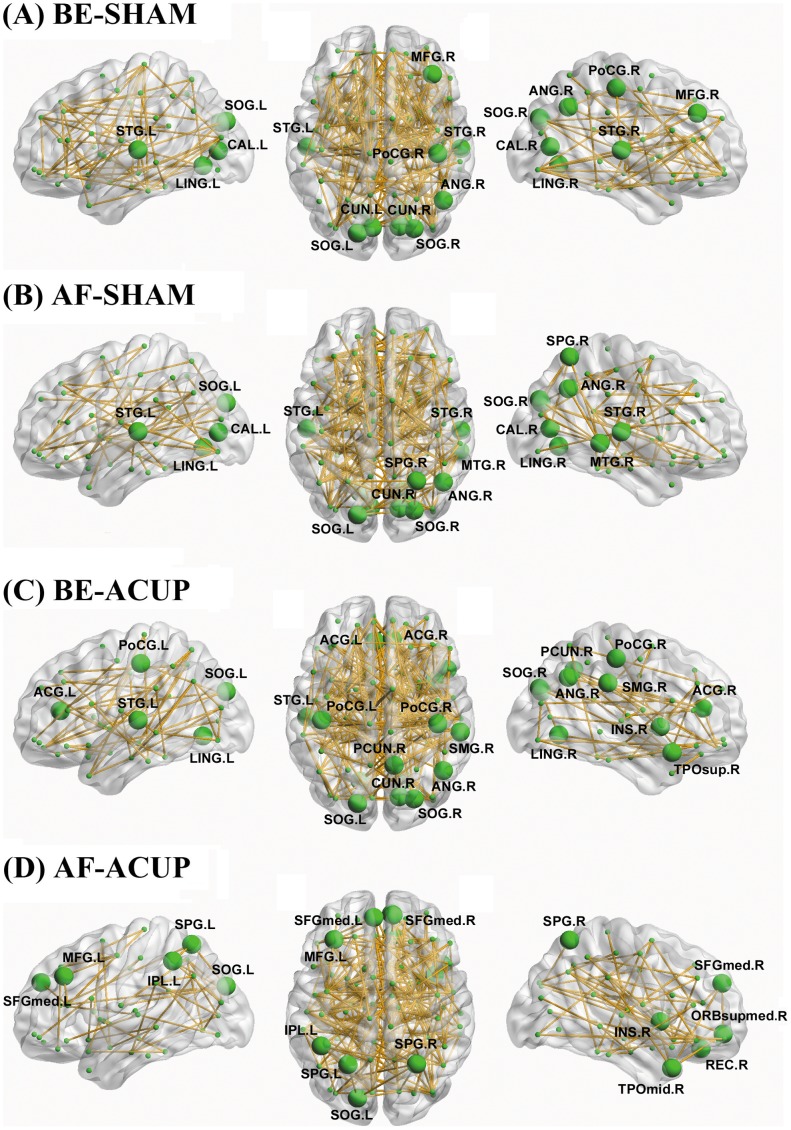
The hubs of functional brain networks. The nodal sizes indicate their relative nodal degree within each condition. Regions with normalized nodal degree greater than mean + SD were identified as hubs. Note that the connectivity backbone (sparsity  = 5%) was obtained by thresholding the mean correlation matrix under each condition. For more details, see Table 2. L, left; R, right.

In the current study, hubs were defined as those regions with one standard deviation larger than the mean of nodal degree over all regions. The hubs identified under each condition were listed in Table 2 and mapped onto brain surface for visualization (Fig. 4). With the exception of AF-ACUP, we found that the hubs were predominately located in the occipital, parietal and temporal lobes, such as the bilateral lingual gyrus (LING), right angular gyrus (ANG) and left superior temporal gyrus (STG). Moreover, we found that the spatial distributions of hub regions were similar among conditions of BE-SHAM, AF-SHAM and BE-ACUP, but changed a lot under AF-ACUP condition. Several frontal regions (e.g., bilateral superior frontal gyrus, medial [SFGmed], right superior frontal gyrus, medial orbital [ORBsupmed], left middle frontal gyrus [MFG] and right gyrus rectus [REC]) became hubs and several occipital regions (e.g., bilateral LING and left STG) no longer served as hubs at AF-ACUP.

**Table 2:** The hubs in the brain functional networks.

Region	Classification	Nodal degree	Region	Classification	Nodal degree
**BE-SHAM**			**BE-ACUP**		
LING.R	Occipital	1.47	LING.L	Occipital	1.36
LING.L	Occipital	1.43	PoCG.R	Parietal	1.27
CAL.R	Occipital	1.39	STG.L	Temporal	1.27
SOG.R	Occipital	1.37	INS.R	Insula	1.26
SOG.L	Occipital	1.36	LING.R	Occipital	1.25
STG.L	Temporal	1.32	CUN.R	Occipital	1.24
CAL.L	Occipital	1.31	SOG.L	Occipital	1.23
STG.R	Temporal	1.30	SMG.R	Parietal	1.22
ANG.R	Parietal	1.26	TPOsup.R	Limbic	1.22
PoCG.R	Parietal	1.25	PCUN.R	Parietal	1.22
MFG.R	Frontal	1.23	ANG.R	Parietal	1.21
CUN.L	Occipital	1.23	ACG.R	Limbic	1.21
CUN.R	Occipital	1.22	ACG.L	Limbic	1.19
			PoCG.L	Parietal	1.19
			SOG.R	Occipital	1.19
**AF-SHAM**			**AF-ACUP**		
STG.R	Temporal	1.44	SFGmed.L	Frontal	1.34
STG.L	Temporal	1.32	MFG.L	Frontal	1.33
LING.R	Occipital	1.31	SOG.L	Occipital	1.26
LING.L	Occipital	1.26	SPG.L	Parietal	1.23
CAL.R	Occipital	1.22	ORBsupmed.R	Frontal	1.23
SOG.R	Occipital	1.20	REC.R	Frontal	1.22
CUN.R	Occipital	1.20	SPG.R	Parietal	1.22
ANG.R	Parietal	1.20	IPL.L	Parietal	1.21
SPG.R	Parietal	1.19	SFGmed.R	Frontal	1.21
MTG.R	Temporal	1.18	TPOmid.R	Limbic	1.21
CAL.L	Occipital	1.18	INS.R	Insula	1.20
SOG.L	Occipital	1.18			

Regions were considered hubs if their normalized nodal degree was at least one standard deviation greater than the average across all regions. For the abbreviation of brain regions, see Table 1.

#### Point and stimulation effects

**Figure 5:**
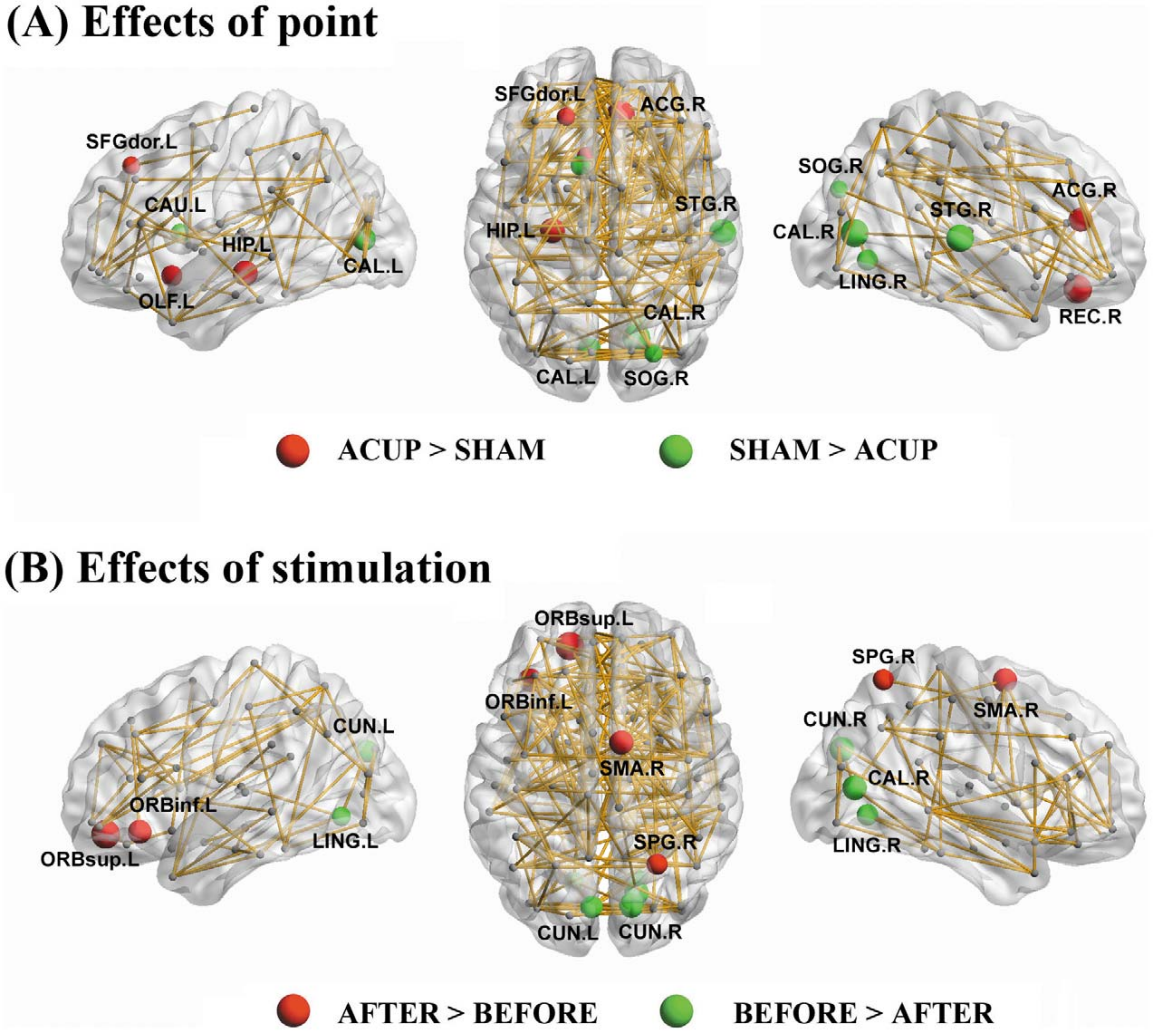
Regions showing significant point- (A) and stimulation- (B) related differences in regional nodal degree. The node sizes indicate the effects (i.e., t values) of interest on nodal degree. The threshold was *P*<0.05 (uncorrected). Note that the connectivity backbone (sparsity  = 5%) was obtained by thresholding the mean correlation matrix across all subjects and conditions. For more details, see Table 3. ACUP, acupuncture at ST36; SHAM, acupuncture at sham point; Before, before stimulation; After, after stimulation; L, left; R, right.

Two-way repeated-measures ANOVA revealed that only the left hippocampus (HIP) exhibited significant (*P*<0.05, false discovery rate [FDR] corrected) point main effect. No regions showed significant stimulation main effect and interaction under this significance level. Post-hoc paired t-tests indicated higher nodal degree of the left HIP at ACUP as compared to SHAM. To further illustrate relatively subtle effects of point and stimulation on nodal centrality, we listed and mapped those brain regions reaching a less rigorous significance level of *P*<0.05 (uncorrected) (Table 3 and Fig. 5). Another 10 brain regions showed point main effects, including 5 regions with increased nodal degree (right REC, right anterior cingulate and paracingulate gyri [ACG], left olfactory cortex [OLF] and left superior frontal gyrus, dorsolateral [SFGdor]) and 6 regions with decreased nodal degree (bilateral calcarine fissure and surrounding cortex [CAL], right STG, left caudate [CAU], right LING and right superior occipital gyrus [SOG]) at ACUP as compared to SHAM. As for stimulation main effects, 4 regions showed increased nodal degree (left superior frontal gyrus, orbital part [ORBsup], right supplemental motor area [SMA], left inferior frontal gyrus, orbital part [ORBinf] and right superior parietal gyrus [SPG]) and 5 regions showed decreased nodal degree (bilateral cuneus [CUN], right CAL and bilateral LING) after the stimulation. Interestingly, all the decreased regions of nodal degree after acupuncture stimulation were located in occipital lobe and were identified as hubs in the brain network (Table 3).

**Table 3:** Effects of interest on regional nodal degree.

Regions	Classification	F(1,17)	Effect	Hub
**Main effect of point**				
HIP.L	Limbic	6.66	ACUP>SHAM	No
REC.R	Frontal	13.24	ACUP>SHAM	Yes
ACG.R	Limbic	9.27	ACUP>SHAM	Yes
OLF.L	Frontal	8.00	ACUP>SHAM	No
SFGdor.L	Frontal	4.75	ACUP>SHAM	No
CAL.L	Occipital	12.85	SHAM>ACUP	Yes
CAL.R	Occipital	12.56	SHAM>ACUP	Yes
STG.R	Temporal	11.97	SHAM>ACUP	Yes
CAU.L	Subcortical	6.45	SHAM>ACUP	No
LING.R	Occipital	6.17	SHAM>ACUP	Yes
SOG.R	Occipital	4.94	SHAM>ACUP	Yes
**Main effect of stimulation**				
ORBsup.L	Frontal	14.61	AFTER>BEFORE	No
SMA.R	Frontal	6.56	AFTER>BEFORE	No
ORBinf.L	Frontal	6.42	AFTER>BEFORE	No
SPG.R	Parietal	4.78	AFTER>BEFORE	Yes
CUN.R	Occipital	10.84	BEFORE>AFTER	Yes
CAL.R	Occipital	10.76	BEFORE>AFTER	Yes
LING.R	Occipital	8.23	BEFORE>AFTER	Yes
CUN.L	Occipital	8.09	BEFORE>AFTER	Yes
LING.L	Occipital	5.13	BEFORE>AFTER	Yes

R, right hemisphere; L, left hemisphere. The effects of points and stimulation were determined by two-way repeated-measures ANOVA. The directions of the effect of interest were determined by post-hoc paired t-tests. The threshold was *P*<0.05 (uncorrected). Only the HIP.L survives after the multiple comparison correction (*P*<0.05, FDR corrected). “Yes” indicates that the region was identified as a “hub” of any of the four conditions of networks and “No” indicates that the region was not a hub. For the abbreviation of brain regions, see Table 1.

### Regional Nodal Homogeneity

**Figure 6:**
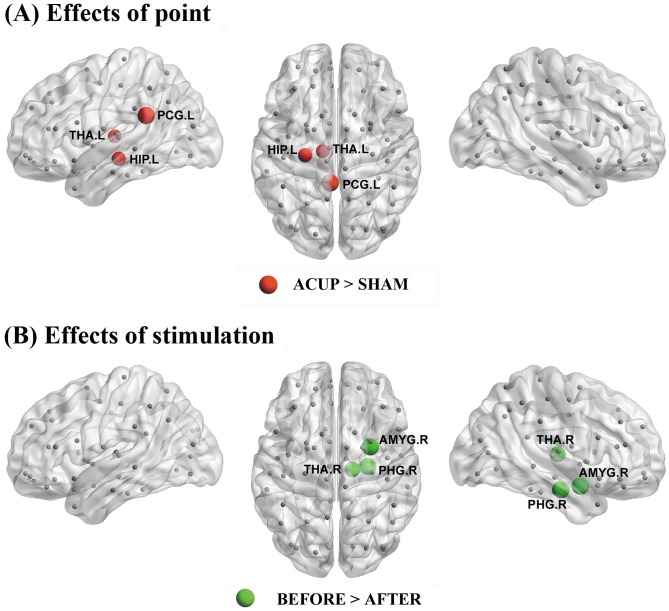
Regions showing significant point- (A) and stimulation- (B) related differences in regional nodal homogeneity. The node sizes indicate the effects (i.e., t values) of interest on nodal degree. The threshold was *P*<0.05 (uncorrected). Note that the connectivity backbone (sparsity  = 5%) was obtained by thresholding the mean correlation matrix across all subjects and conditions. For more details, see Table 4. ACUP, acupuncture at ST36; SHAM, acupuncture at sham point; Before, before stimulation; After, after stimulation; L, left; R, right.

In addition to the abovementioned network metrics based on interregional functional connectivity, we also calculated intraregional homogeneity for each brain area [40]. No brain regions exhibited significant (*P*<0.05, FDR corrected) point and stimulation effects. Under an uncorrected *P*<0.05, several limbic and subcortical brain regions were found to be relatively sensitive to acupuncture point (increased nodal homogeneity in the left HIP, left posterior cingulate gyrus [PCG] and left thalamus [THA] at ACUP as compared to SHAM) and simulation (decreased nodal degree in the right parahippocampal [PHG], right amygdala [AMYG] and right THA after the stimulation) (Table 4 and Fig. 6).

**Table 4:** Effects of interest on regional nodal homogeneity.

Regions	Classification	F(1,17)	Effect	Hub
**Main effect of point**				
HIP.L	Limbic	6.66	ACUP>SHAM	No
PCG.R	Limbic	5.68	ACUP>SHAM	No
THA.L	Subcortical	4.65	ACUP>SHAM	No
**Main effect of stimulation**				
PHG.R	Limbic	8.04	BEFORE>AFTER	No
AMYG.R	Subcortical	6.20	BEFORE>AFTER	No
THA.R	Subcortical	4.58	BEFORE>AFTER	No

R, right hemisphere; L, left hemisphere. The effects of points and stimulation were determined by two-way repeated-measures ANOVA. The directions of the effect of interest were determined by post-hoc paired t-tests. The threshold was *P*<0.05 (uncorrected). No regions survive after the multiple comparison correction (*P*<0.05, FDR corrected). “Yes” indicates that the region was identified as a “hub” of any of the four conditions of networks and “No” indicates that the region was not a hub. For the abbreviation of brain regions, see Table 1.

## Discussion

This is the first study to investigate the small-world properties of brain functional networks in acupuncture at ACUP and SHAM and between states before and after acupuncture. We found that brain functional networks exhibited efficient small-world topology under each condition. However, increased local efficiency in brain functional networks was demonstrated only after stimulating acupuncture point ST36. Furthermore, our study revealed that nodal degree was profoundly affected at several regions of the limbic system, prefrontal, parietal, temporal and occipital cortices, a finding that is compatible with previous studies in acupuncture. Our results suggested that the topological organization of functional brain networks is altered after acupuncture and the alterations have point specificity, thus providing further evidence for brain modulation associated with point and stimulation.

In the current study, we utilized a specific connectivity analysis method, graph-based network analysis, to explore the acupuncture mechanism. Currently, several connectivity approaches exist, including ReHo (23), seed-based connectivity analysis (24) and ICA (25). ReHo quantifies the similarity of the time series of a given voxel to those of its nearest neighbors, thus only measuring the relationships between the given voxel and those spatially adjacent voxels without taking into account long-range connections. Seed-based approach measures functional connectivity between a specific region of interest (ROI) and all the other voxels in the brain and thus it only takes into account of the connectivity relevant to the ROI without taking into account of the relationships among other regions. ICA attempts to identify sets of brain regions that are separable on the basis of statistical patterns in their dynamic time series, thus providing information about how regions may be related within subnetworks (i.e., components) but not capturing the connectivity information between these components. Although these connectivity methodologies have been successfully applied to map brain connectivity networks from different perspectives and revealed disease-related alterations in these networks, none of them can capture the topological structure of these brain networks. In contrast, graph theoretical approaches allow us to map the entire functional connectivity pattern among all the brain units simultaneously and to explore how the layout is organized and modulated in response to external stimulus, such as acupuncture. Moreover, studying the full connectivity network at a system level conforms to conditional view that acupuncture modulates and balances the brain activity from global rather than local levels [26], [27].

Previous researches have shown that human brain functional networks follow a small-world configuration [30], [33]. In agreement with the previous findings, in the present study, we also observed the features of small-world architecture in the functional brain networks during acupuncture at ACUP and SHAM between before and after acupuncture. Moreover, the functional networks showed economical properties, which was in accordance with previous human brain structural and functional networks studies [41], [42], [43].

The topology of after acupuncture signal was altered as compared to the before acupuncture signal. The result showed an after-larger-than-before local efficiency at ACUP but non-significant differences at SHAM. The local efficiency is a measure of local network connectivity. Previous studies have shown that the connectivity of distinct brain regions can be modulated by acupuncture. For example, Qin et al. [6] indicated that acupuncture can increase connectivity between amygdala with other brain regions including the medial prefrontal cortex, postcentral gyrus, insula and periaqueductal gray. Dhond et al. [36] reported that acupuncture increased not only the default mode network (DMN) connectivity with pain, affective and memory related brain regions, but also the sensorimotor network (SMN) connectivity with pain-related brain regions. These alterations may contribute to the increasing tendency of local efficiency in the brain network after acupuncture. We thus suspected that the higher value of local efficiency after acupuncture observed here might suggest a kind of regional reorganization mechanism in response to an external stimulus.

The regional nodal degree measures the extent to which a given node connects all other nodes of a network and indicates the importance or centrality of the node in the whole-brain network. Using this measure, we found several occipital (e.g., the LING, CUN and SOG), temporal (e.g., the STG) and parietal (e.g., the PCUN, ANG and PoCG) regions exhibited high nodal degree and therefore were considered hubs. These hubs were highly consistent among conditions of BE-SHAM, BE-ACUP and AF-SHAM and many of them were identified as functional and/or structural core regions in previous studies [29], [32], [44], [45], [46], [47], [48], [49]. Nonetheless, we noted that there were still several regions that were detected as hubs previously but not in the current study, such as medial prefrontal cortex [45]. These discrepancies could be due to different preprocessing strategies, sample characteristics and nodal metrics employed by these studies. As for AF-ACUP, hubs regions were predominantly located in frontal and parietal lobes, suggesting a redistribution of hubs regions, which presumably is due to the modulation of ACUP.

Further statistical analysis revealed altered regional nodal degree centrality associated with point-related effects in several regions, involving the limbic system (e.g., right ACG and left HIP), temporal (right STG), occipital (bilateral CAL, right LING and right SOG) and frontal (left SFGdor, left OLF and right REC) regions. Compared with SHAM, ACUP induced increased regional nodal degree in the ACG, HIP and frontal regions, and decreased nodal centrality in the temporal and occipital cortices. These findings are compatible with previous functional imaging studies [7], [12], [13], [16] and suggest distinct modulation mechanisms recruited by ACUP and SHAM in regulating nodal centrality. According to traditional Chinese medicine, ACUP point is a specific anatomical position of the human body, but SHAM point is not. Subjects receiving stimulations at ACUP point usually generate sensations of DeQi (a series of unique sensations of numbness, tingling, fullness, and dull ache that develop at the site of acupuncture). Although the sensations of DeQi could also appear at SHAM point, there are more varied and stronger sensations evoked by needling at ACUP as compared to SHAM points [30]. Previous functional MRI studies have showed point-related differences in the neural activities induced by acupuncture between at ACUP and at SHAM points [10], [13], [30]. Our findings of point-related effects on regional nodal centrality reported here indicate that the nodal centralities in brain functional networks are profoundly affected by different points, therefore providing further evidence of acupoint specificity. However, it is worth noting that we did not find significant between-group differences in DeQi for the samples employed in the current study, which could be due to the relatively small sample size. Further large sample studies are needed to provide more insights into this issue.

In this study, we also showed altered regional nodal degree centrality associated with stimulation-related effects in several regions, including the frontal (left ORBsup, right SMA and left ORBinf), parietal (right SPG) and occipital (right CAL, bilateral LING and CUN) regions. The left ORBsup, left ORBinf and right SPG are located in the frontal-partial circuit, which is associated with the attention and executive function network [50]. Increased nodal centrality in the frontal and parietal cortex after needle stimulation was consistent with several functional imaging studies that have found activation in these regions [51], [52], which might suggest the enhancement of attention and executive function after needle stimulation. In contrast, several regions belonging to occipital cortices were also found to exhibit decreased nodal centrality. Evidence shows that stimulation of human frontal [53] and parietal [54] regions can affect visual cortical activity. When increasing load of attention and executive function, visual cortical activity became attenuated [55]. Our results showed increased nodal centrality in frontal and parietal regions and decreased nodal centrality in occipital regions, which are compatible with a previous study showing acupuncture-related functional alteration in these regions [56]. Together, the findings of stimulation-related changes in the regional nodal centrality reported here suggest that the nodal roles in brain functional networks are profoundly affected by needle stimulation. Further, we suspect that the cooperation among frontal, parietal and occipital cortex regions could contribute to the effect of acupuncture.

In addition to stimulation- and point-related alterations on nodal degree that measures the extent to which a given node connects to other nodes in a network, we also found that several limbic and subcortical brain regions were modulated with respect to their local homogeneity. It should be noted that after multiple comparison correction, significant modulation was observed only for nodal degree, implying the regulation of acupuncture mainly on interregional connectivity.

Several issues need to be further addressed. First, the subjects in our study were healthy individuals, but previous studies found that acupuncture was an effective treatment for pathological conditions (e.g., Parkinson’s disease). Thus, studying the effects of acupuncture under different conditions of clinical diseases would be an interesting subject for future studies. Second, we constructed whole-brain functional networks and investigated their topological attributes in responding to acupuncture. Several previous studies suggest that the topological behavior of specific brain subsystems or functional modules is different from that of the whole network [29], [57]. Consequently, the investigation of specific sub-networks associated with acupuncture (e.g., pain-related networks) would provide further insights into the mechanism of acupuncture. Finally, the nodal centrality results reported in the current study were not corrected by multiple comparisons, meaning this finding needs to be considered an exploratory analysis. Future studies using a large sample of participants or selecting a limited number of ROIs can increase the statistical power.

To summarize, we evaluated the stimulation- and point-related differences in human brain functional networks based on fMRI. Our results indicate that the topological organization of human brain functional networks can be modulated by different acupuncture points and stimulations. Together, our results indicate that graph theoretical network analysis could provide an important tool to explore the mechanism of acupuncture.

## Methods

### Subjects

The experiment was performed on 18 healthy, right-handed Chinese college students (9 males and 9 females; aged  = 23∼27 years, mean age  = 25.1 years, SD  = 2.83 years; mean education level  = 15.28 years, SD  = 2.19 years). Subjects with a medical history of any neurological or psychiatric conditions were excluded from the study. All subjects were naïve to acupuncture and had not been previously exposed to a high magnetic field. Written informed consent was obtained from each participant, and the study was approved by the ethics committee of the Second Affiliated Hospital, Guangzhou University of Traditional Chinese Medicine.

### Experimental Design

The experiment lasted a total of 57 minutes and was composed of an initial rest scan of 6 minutes, 20 minutes of acupuncture treatment (actual or sham), a 25-minute resting period after the needle was removed, and a 6-minute post-acupuncture scan (Fig. 1).

Acupuncture was performed at the acupoint ST36 (zusanli, located four finger width below the lower margin of the patella and one finger width laterally from the anterior crest of the tibia) using disposable sterile needles (40 mm long ×0.30 mm diameter supplied by Huatuo, Suzhou Medical Application Company). The needle was inserted perpendicularly to the skin surface to a depth of 15 mm. In the ACUP, the needle was manipulated rotationally with a flipping range of ±180° at a frequency of 120 flips/min for 1 min at 0 minutes, 7 minutes and 14 minutes. The total needle retention time was twenty minutes per run. In the SHAM, acupuncture was carried out with needling at non-meridian points (2–3 cm away from ST36) with needle depth, stimulation intensity, and manipulation identical to that of ACUP group. The procedure was performed by the same experienced and licensed acupuncturist on all subjects.

Most of the acupuncture studies use unilateral acupoint only with a short needle retention time (a few minutes) without any needle rotation. In the actual clinical practice of acupuncture, it is usually performed on both limbs with needle retention time ranging from 20 to 30 minutes along with needle rotation at specified time intervals. In this study we have emulated the clinical practice of acupuncture in its entirety and we thus presume that the results would truly reflect the clinical effects of acupuncture.

All 18 subjects were divided into two groups, each with 9 subjects: one group received ACUP, whereas the other received SHAM, and the groups were alternated each week. To eliminate the anticipatory effects of the acupuncture, the presentation sequence of these two runs (SHAM and ACUP) was randomized and each participant was subjected only once each week. The subjects were not informed of the order in which the two runs would be carried out, and they were instructed to remain tranquil without engaging in any mental task. To facilitate blinding, the subjects were also asked to keep their eyes closed to prevent them from actually observing the procedures. At the end of each scan, all subjects were questioned to confirm that they had stayed awake during the entire process.

### Image Acquisitions and Data Preprocessing

The fMRI data were obtained using a 1.5 T Siemens scanner at the department of radiology of the Second Affiliated Hospital of Guangzhou University of Traditional Chinese Medicine. A total of 180 volumes of EPI images were obtained axially (repetition time, 2000 ms; echo time, 30 ms; slices, 30; thickness, 4 mm; gap, 1 mm; field of view, 240×240 mm^2^; matrix, 64×64; flip angle, 90°). Prior to preprocessing, the first 5 volumes were discarded to allow for scanner stabilization and the subjects’ adaptation to the environment. Data preprocessing was then conducted by SPM5 (http://www.fil.ion.ucl.ac.uk/spm/). Briefly, the remaining functional scans were first corrected for within-scan acquisition time differences between slices, and they were then realigned to the first volume to correct for inter-scan head motions. This realigning step provided a record of head motions within each fMRI run. One subject was found to have excessive head motion (larger than 2 mm and/or 2 degree in any direction) and was therefore excluded from further analysis. Subsequently, the functional scans were spatially normalized to a standard Montreal Neurological Institute space with the EPI image provided by SPM5 as a reference template and resampled to 3×3×3 mm^3^ resolution. Finally, the waveform of each voxel was passed through a band-pass filter (0.01–0.1 Hz) to reduce the effects of low-frequency drift and high-frequency physiological noise.

### Construction of Functional Brain Networks

In the current study, functional brain networks were constructed for each participant with nodes denoting brain regions and edges denoting functional connectivity between nodes. To define network nodes, we employed an automated anatomical labeling (AAL) atlas [58] to divide the brain into 90 ROIs (45 in each hemisphere). The names of the ROIs and their corresponding abbreviations are listed in Table 1. The mean time series was then acquired for each ROI by averaging the signals of all voxels within that region. Prior to the interregional functional connectivity estimation, multiple linear regressions were performed to remove several sources of spurious variances arising from estimated head-motion profiles and global activity from each regional mean time course [59]. Pearson correlation coefficients between any pair of regional residual time series were subsequently calculated, thus generating a 90×90 correlation matrix for each subject. Finally, each absolute correlation matrix was thresholded into a binary matrix with a fixed sparsity level, S (defined as the total number of edges in a network divided by the maximum possible number of edges). Setting a sparsity threshold ensured that all the resultant networks had the same number of edges or wiring cost. Given that there is currently no definitive way to determine a single threshold level, we thresholded each absolute correlation matrix repeatedly over a wide range of sparsity levels (10%≤S≤40%) at an interval of 0.01. This range of sparsity was chosen to allow prominent small-world properties in brain networks to be observed [32], [43]. Through this thresholding procedure, a set of unweighted and undirected graphs were obtained for each subject. See Figure 1C for the flowchart of brain network construction.

### Network Analysis

#### Small-world analysis

The small-world model, originally proposed by Watts and Strogatz [60], can be quantified by characteristic path length, 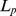 and clustering coefficient, 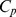. In a small-world network, the shortest path length between any pair of nodes was approximately equivalent to a comparable random network, but the nodes of the network had greater local interconnectivity than a random network [60]. Recently, more biologically relevant measures of network efficiency have been widely used to characterize the capability of parallel information flow in functional brain networks [29], [34], [41], [61]. Network efficiency provides a single measure to capture both the local and the global behavior of a network and can also address either disconnected or non-sparse graphs or both [62]. In this study, we used the efficiency measures to investigate the effects of acupuncture and differences between actual and sham acupoints in functional brain functional networks. Briefly, for a graph 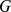 with N nodes and K edges, the global efficiency was defined as
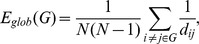
where 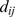 is the shortest path length between node 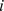 and node 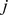 in 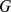. The local efficiency of 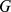 was measured as



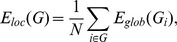
where 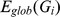 is the global efficiency of 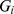, the sub-graph comprised of the neighbors of node 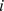. Global efficiency and local efficiency measure the ability of a network to transmit information at the global and local level, respectively [62]. In this study, we also investigated the ratios of local efficiency (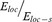) and global efficiency (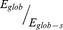) between the real brain functional networks and 100 random networks to assess small-world properties of functional brain networks. The random networks were generated by matching the number of nodes and edges as well as degree distribution with actual brain networks [63], [64]. Typically, a small-world network has a higher local efficiency (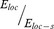
>1) and an approximately equivalent global efficiency (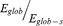
≈1) as compared to its random counterparts.

#### Nodal centrality

To examine the regional properties of brain functional networks, we employed nodal degree among numerous nodal metrics because of its high test-retest reliability [65]. For a given node 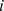, the nodal degree 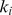 measures the connectivity of this node with all the other nodes in a network and is calculated as the number of edges linked to it.

In this study, all the network metrics used (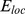, 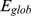 and 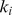) were functions of the sparsity threshold because functional brain networks were constructed over a continuous threshold level (10% ≤ S ≤40%). To provide a summarized scalar for each metric and simplified subsequent statistical analysis, we calculated the integrated global efficiency, local efficiency and nodal degree as areas under curves (AUCs) for each subject, which has been used in previous brain network studies [29], [34], [41].

In addition to the abovementioned network metrics based on interregional functional connectivity, we also calculated intraregional homogeneity in the time series fluctuations for each brain area [40]. For a region or node, the intraregional homogeneity is calculated as.
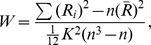
where 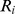 is the sum rank of the *i*th time point; 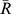 is the mean of 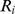; 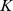 is the number of voxels in the region; and 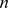 is the length of the time series.

### Statistical Analysis

To determine whether there were significant differences in any of the global efficiency, local efficiency, nodal degree and nodal homogeneity, two-way repeated-measures analysis of variance (ANOVA) was performed on the these metrics (AUCs) with points (ACUP and SHAM) and stimulation time (before and after stimulation) as within-subject factors. The ANOVA was performed by (http://www.mathworks.com/matlabcentral/fileexchange/6874-two-way-repeated-measures-anova).

### Acupuncture Sensation Analysis

At the end of each fMRI scan, all subjects completed a questionnaire based on a 10-point visual analog scale (VAS) to rate their experience of any pain sensation (sharp, full, dull), soreness, numbness, fullness, heaviness, throbbing, warmth, coolness and any other sensations experienced during the scan [7], [66]. The VAS was scaled at 0 =  no sensation, 1–3 =  mild, 4–6 =  moderate, 7–8 =  strong, 9 =  severe, and 10 =  unbearable sensation. Sharp pain was considered to be an inadvertent noxious stimulation [66]. Fisher’s exact test and Student’s t test were used to compare the frequency and the intensity of sensations between ACUP and SHAM. Subjects who experienced sharp pain (greater than two standard deviations above the mean pain level) were excluded from further analysis.
